# Population genetic structure of Randall’s threadfin bream *Nemipterus randalli* in Indian waters based on mitochondrial and nuclear gene sequences

**DOI:** 10.1038/s41598-024-58047-1

**Published:** 2024-03-30

**Authors:** Neenu Raj, Sandhya Sukumaran, Anjaly Jose, K. Nisha, Subal Kumar Roul, Shikha Rahangdale, Shoba Joe Kizhakudan, A. Gopalakrishnan

**Affiliations:** 1 Marine Biotechnology Fish Nutrition and Health Division, ICAR-Central Marine Fisheries Research Institute, Ernakulam North P.O., Kochi, Kerala 682018 India; 2 Mangalore University, Mangalagangotri, Mangalore, Karnataka 574 199 India

**Keywords:** Genetic markers, Population genetics

## Abstract

*Nemipterus randalli*, commonly known as Randall’s threadfin bream, is a commercially important marine finfish. Understanding its genetic structure is critical to effective management and conservation efforts. Previous investigations on population structure in this species were limited by geographic coverage. In this study, we utilized the mitochondrial Cytochrome b gene and nuclear Ribosomal protein gene intron Rp S7 sequences to investigate the population genetic structure, demography and genetic diversity of *N. randalli* along Indian waters. Our results revealed high haplotype diversity but low nucleotide diversity. AMOVA revealed that the variation among the population was highly significant. Hierarchical AMOVA provided further evidence of significant genetic differentiation between the west and east coasts, which was corroborated by the Bayesian tree and the median-joining network diagram. The mtDNA sequences revealed significant genetic structure between populations based on fixation index analysis following the isolation-by-distance model. Furthermore, the neutrality test and mismatch analysis suggest that *N. randalli* populations may have experienced a population expansion. However, nuclear marker RpS7, showed a high level of polymorphism, which obscured the population structuring observed with the mitochondrial marker. Consequently, concordant results were not obtained when comparing the mitochondrial and nuclear DNA sequences. The strong genetic differentiation between the east and west coast observed using mitochondrial marker could be attributed to a combination of geographic and environmental factors. These findings lay the groundwork for developing effective conservation and management strategies for *N. randalli*, considering its genetic structure.

## Introduction

Nemipterids are marine, brightly coloured, small to medium-sized fishes distributed along tropical and sub-tropical Indo-Pacific regions. The family Nemipteridae consists of five genera and 71 species, which constitute a major component of commercial fisheries. Among the *Nemipterus* genera, *Nemipterus randalli* is one of the most commonly caught species in demersal landings (38.1%) and has been targeted by hook and line and bottom trawlers^1^. Randall’s threadfin bream, *Nemipterus randalli*, is one of the most commercially important demersal finfish species in Indian waters due to its edibility and export value. It provides raw materials for the production of surimi, fish balls and fish cakes^2^, which are important for export^3^. It is a non-migratory fish that inhabits the muddy and sandy bottoms in coastal waters at a depth range of 22–450 m, usually in schools. It is distributed in the western Indian Ocean including India, Pakistan, Persian Gulf, Red Sea, Gulf of Aden, East African coast, Seychelles and Madagascar^4^. *N. randalli* is categorized as the least concern species in the IUCN Red List of Threatened Species^5^ due to its wide distribution. They are fractional spawners with protracted spawning periods^6^ with an average life span of about 3 years^7^. They are demersal carnivores which feeds on crustaceans, squids, annelids and small fishes^8^ and ecologically plays a key role in trophic level as secondary consumers (trophic level 3.8). In the recent years, the annual catch of threadfin bream in India was accounted for 81,000 tonnes during 2019–2020^9^. An increase in the production of *N. randalli* could potentially suggest a rise in fishing activities driven by a growing market demand. However, if not managed properly, this could lead to overfishing and depletion of local stocks.

To effectively manage a fishery, it is crucial to have a clear understanding of the stock structure of the species. This understanding is essential for developing appropriate management regulations, particularly in cases where multiple stocks are subject to varying levels of exploitation^10^. Fisheries stock assessments rely heavily on stock identification, which is crucial for effective management of fish populations. For effective management, various stock-rebuilding strategies must be implemented that involves identifying the stock structure^11^. Marine fish species often exhibit weak genetic differentiation due to the lack of physical barriers along with high gene flow and dispersal rate^12^. Especially in migratory fish species, the signal of genetic differentiation is weak and it is difficult to delineate the population structure. With the advent of improved genetic methods, distinct populations or stocks can be resolved. Although there are no apparent physical barriers to gene flow, population structure is often detected in many marine species^13^. This may arise due to various factors such as reproductive isolation, geographical distance^14^, biogeographic barriers^15^, or specific behavioural traits^16^. The advent of molecular markers has proven to be a valuable tool for detecting genetic variation in fish populations at both inter-specific and intra-specific levels. This technology is so sensitive that even low levels of differentiation can be detected. One such marker is mitochondrial DNA (mtDNA), which can be used as an effective marker to elucidate genetic structure due to its rapid evolutionary rate, uniparental inheritance, high mutation rates and lack of recombination^17^. Another set of markers are nuclear EPIC markers, which are extremely effective in detecting genetic structure^18^ due to their high polymorphism^19^.

Understanding the population structure and genetic diversity of this commercially important species is crucial for sustainable utilisation, conservation and management. To obtain deeper insights into the genetic diversity and population structure, genetic tools such as mitochondrial DNA and nuclear DNA have been widely used^20^. In this study, we used molecular markers with different rates of evolution, to investigate the population structure and genetic diversity of *N. randalli* from Indian waters. Mitochondrial DNA markers such as Cytochrome b sequences have proven to be highly effective for studying diversity and systematics. These markers exhibit a combination of slowly and rapidly evolving codon positions, as well as conserved and variable regions. This unique property makes Cytb DNA sequences ideal for investigating a wide range of questions, including phylogeny, population dynamics, and recent divergence levels^21^. To assess genetic variability, nuclear DNA markers such as Ribosomal protein Exon primed Intron Crossing (Rp EPIC) were used, which span the exon regions and detect polymorphisms within the conserved intron region and can be used as selectively neutral nuclear markers^22^.

Despite its commercial importance, there is little of information about the population structure of *N. randalli* in Indian waters. Studies on *N. randalli* from Indian waters are limited to stock assessment^1^, biology^23^, diversity^24^, morphometrics^25^ and systematics^26^. A recent investigation of *N. randalli* based on mitochondrial D-loop makers indicated lack of stock structuring in Indian waters^27^. However, the same authors reported the stock structure in Indian waters based on morphological parameters and otolith shape^28^. However, these studies were fragmented and incomplete. We conducted a comprehensive investigation by collecting samples along the entire Indian coast and assessing the genetic structuring using mitochondrial and nuclear markers.

We used two classes of genetic markers, the mitochondrial Cytochrome b (Cyt b) gene and the nuclear Ribosomal protein gene intron S7 (Rp S7). We also investigated the historical demography of *N. randalli* in Indian waters, which is critical for proper management and ensuring sustainability of the fishery.

## Results

### Mitochondrial DNA

A 507 bp of Cytb gene sequence was amplified from 125 individuals from five locations. The Cytb sequences had 53 polymorphic sites consisting of 58 substitutions including 47 transitions and 11 transversions. Out of 507 sites, 454 were found to be invariable while 53 sites were variable. Furthermore, there were 28 singleton sites and 25 parsimony informative sites. The average nucleotide composition was C: 32.86%, T: 29.86%, A: 24.63% and G: 12.65%. Point mutations occurred at the first and second codon positions, resulting in seven non-synonymous amino-acid substitutions. Out of 125 individuals, a total of 58 haplotypes were identified, with 51 of them being private haplotypes (87.93%) and the remaining 7 (12.06%) being shared haplotypes (Table S1). While the majority of the private haplotypes were unique to a single individual, two private haplotypes were identified in multiple individuals. Table 1 provides information on the number of individuals, number of haplotypes, segregating sites, haplotype diversity, and nucleotide diversity for each location. Overall haplotype diversity was high (H_d_ = 0.893), but nucleotide diversity was low (π = 0.012) in *N. randalli*.

**Table 1:** Diversity measures of *N. randalli* for Cytb and Rp S7 sequences.

Region	Sampling location	Genetic polymorphism										
		n	mtDNA Cyt b					nuDNA Rp S7				
			S	h	k	H_d_	π	S	A	k	A_d_	π
West coast India	Cochin	25	20	12	2.226	0.816	0.0043	145	41	22.803	0.992	0.039
	Mangalore	25	13	12	1.660	0.696	0.0032	104	35	17.124	0.987	0.029
	Veraval	25	15	13	1.613	0.776	0.0031	163	39	26.125	0.991	0.045
East coast India	Chennai	25	20	18	2.073	0.926	0.004	105	37	14.400	0.989	0.024
	Puri	25	13	11	1.713	0.770	0.0033	154	34	26.685	0.986	0.046

Number of specimens (n), Number of segregating sites (S), Number of haplotypes (h), average number of nucleotide differences (k), haplotype diversity (H_d_), Allelic diversity (A_d_), nucleotide diversity (π).

**Figure 1:**
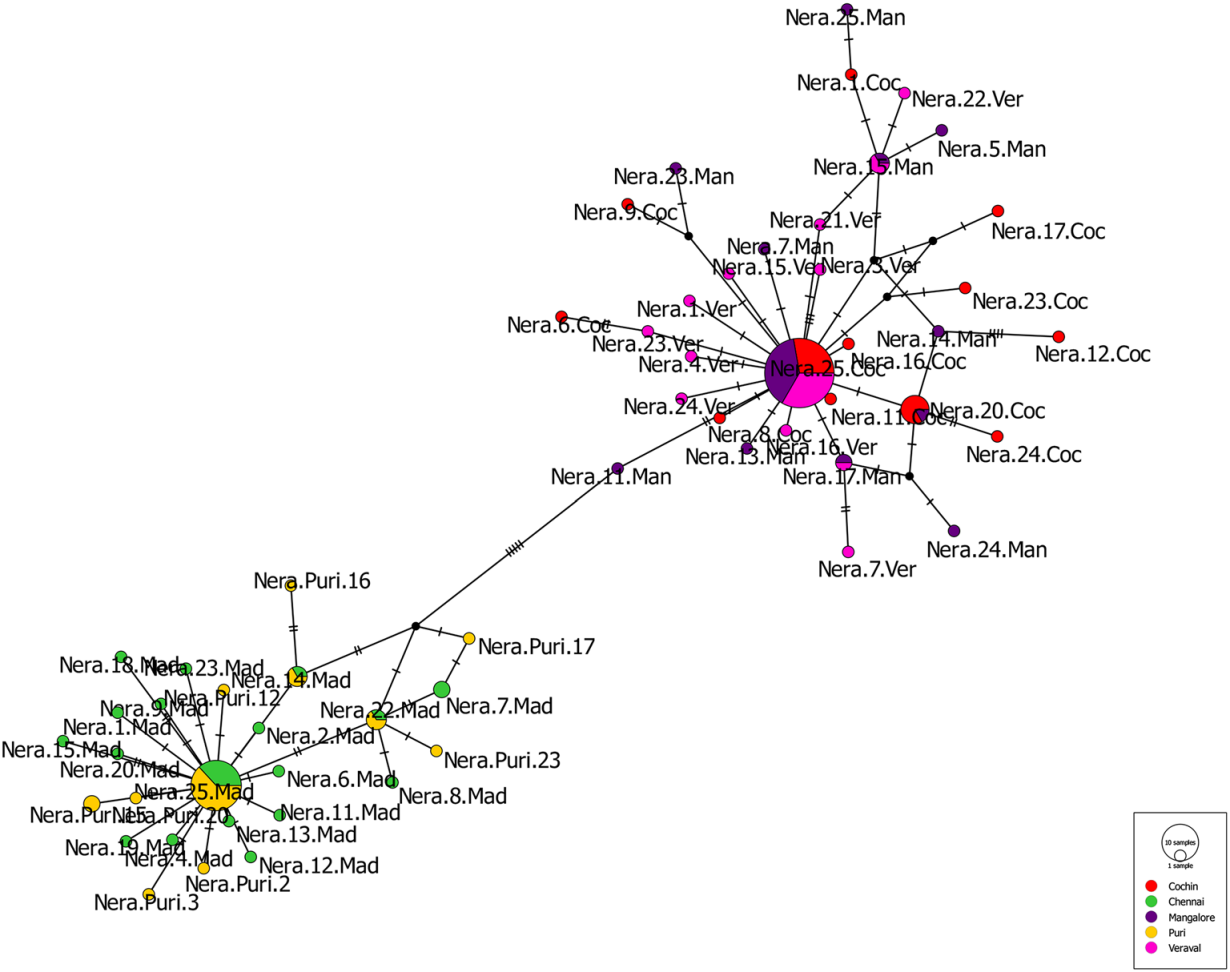
Median joining network for *N. randalli* based on Cytb sequences. Haplotypes are represented in circles and colours indicate geographical locations. Vertical lines indicate mutational steps. Black dots indicate lost or not sampled haplotypes.

Haplotype network analysis using the median-joining method identified two haplogroups, which were separated by five mutation steps as illustrated in the diagram (Fig. 1). The central part of the network was occupied by shared haplotypes, while the unique haplotypes branched out from the centre. Haplogroup I with the common haplotype H13, comprised of haplotypes from the east coast (Chennai and Puri). On the other hand, haplogroup II with a common haplotype of H1, included haplotypes from the west coast (Veraval, Mangalore, and Cochin). The distribution of haplotypes based on Cytb gene sequences exhibited a star-like pattern indicating population expansion.

**Figure 2:**
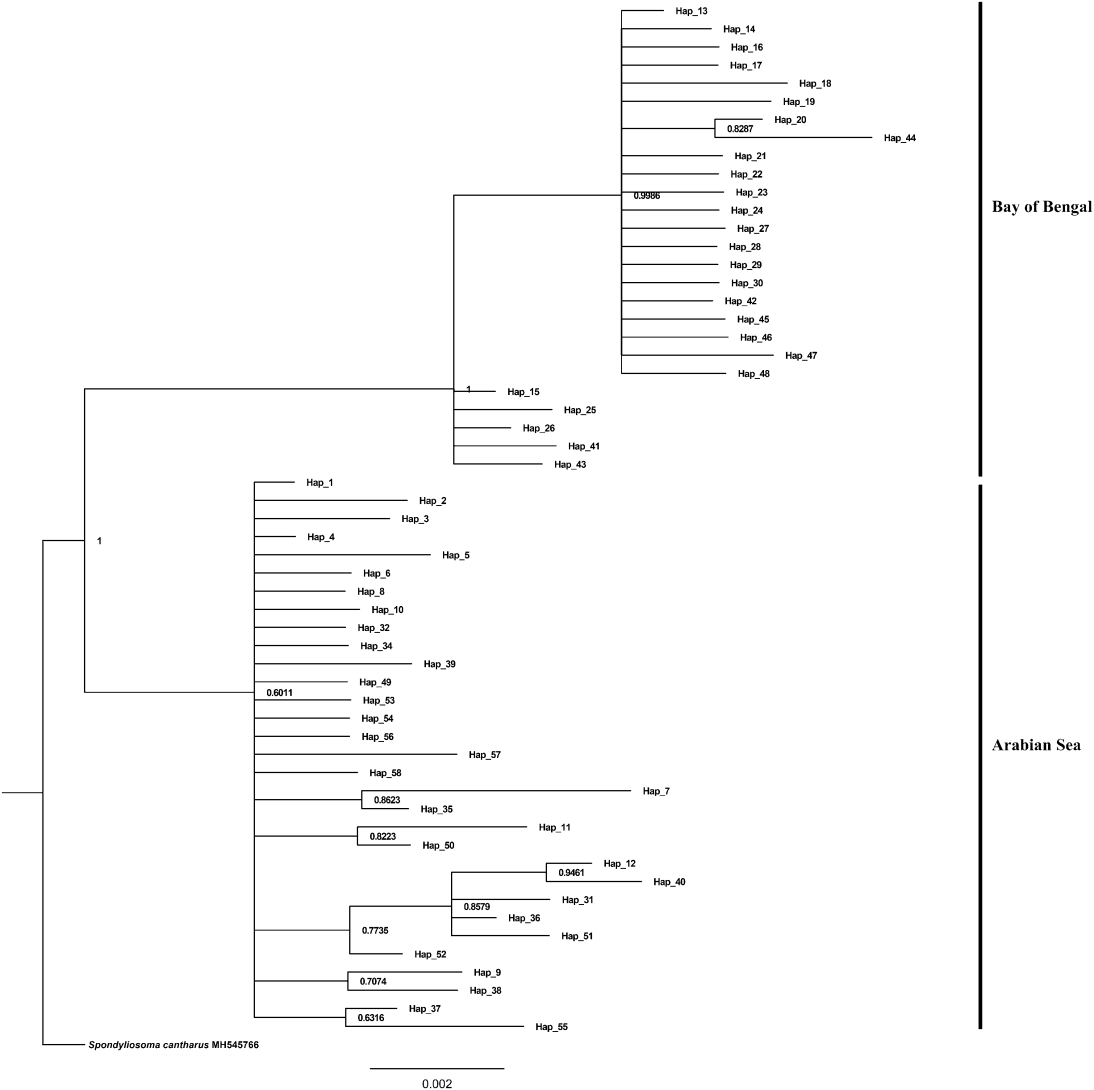
Bayesian tree for Cytb haplotypes of *N. randalli*. Posterior probability values are indicated at the branches.

The range of genetic divergence within the population varied between 0 and 0.2%, with the lowest value observed in Mangalore and Puri, and the highest in Cochin. Genetic divergence between populations ranged from 0.1 to 0.8%. Among the populations compared, Coc-Man, Coc-Ver, Che-Pur, and Man-Ver exhibited the least divergence, while Coc-Che, Coc-Pur, Che-Man, Che-Ver, Man-Pur, and Pur-Ver showed the highest divergence. However, when the populations were categorized according to their respective coasts, the divergence within each coast was only 0.1%, while between coasts, it reached 0.7% (Table 2). Therefore, the K2P distance analysis indicated genetic divergence among *N. randalli* populations along both coasts of India. Bayesian analysis displayed two major clusters. The upper cluster contained haplotypes from the East Coast and the lower branch contained haplotypes from the West Coast with relatively high posterior probability value (Fig. 2).

**Table 2:** Pairwise genetic distance within population (shown in bold along diagonal), between population (above diagonal) and fixation index (F_ST_) (below diagonal) based on Cytb sequences and Rp S7 sequences.

Population	Cochin	Chennai	Mangalore	Puri	Veraval
Cytb					
Cochin (COC)	**0.002**	0.008	0.001	0.007	0.002
Chennai (CHE)	0.80282*	**0.001**	0.007	0.001	0.007
Mangalore (MAN)	0.00444	0.82577*	**0.000**	0.006	0.001
Puri (PUR)	0.81254*	− 0.00967	0.83641*	**0.000**	0.006
Veraval (VER)	0.03459	0.83102*	− 0.00979*	0.84196*	**0.001**
Rp S7					
Cochin (COC)	**0.041**	0.036	0.037	0.049	0.047
Chennai (CHE)	0.07770*	**0.025**	0.029	0.040	0.038
Mangalore (MAN)	0.04359*	0.04456*	**0.030**	0.043	0.041
Puri (PUR)	0.09094*	0.07091*	0.07060*	**0.049**	0.052
Veraval (VER)	0.06816*	0.05273*	0.05255*	0.07419*	**0.047**

*Indicates significant values after Bonferroni correction at p < 0.005.

AMOVA analysis indicates statistically significant population differentiation of *N. randalli* (F_ST_ = 0.739, p < 0.05) along the Indian coast, with a hierarchical AMOVA analysis revealing high genetic differentiation between the east and west coasts (Φ_CT_ = 0.824, p < 0.05) (Table 3). Table 2 shows the pairwise F_ST_ values among five *N. randalli* populations, indicating significant differentiation among most of the compared populations except for a few, namely Coc-Man, Coc-Ver, Che-Puri, and Man-Ver, which showed no statistical significance. The findings indicated that populations along the same coast showed insignificant values, suggesting no genetic differentiation, whereas populations between the west and east coast displayed notable genetic differentiation. Thus, the populations can be divided into two groups: those from the west coast forming one group and those from the east coast forming the other. Analysis using SAMOVA revealed that the genetic differentiation among groups was highest when the number of groups (K) was set to 2, resulting in an F_CT_ value of 0.824 (p < 0.05). The first group consisted of populations from the east coast, including Chennai and Puri, while the second group comprised populations from the west coast, namely Cochin, Mangalore, and Veraval. The Mantel test supports isolation by distance, showing a significant positive correlation between genetic and geographical distances (r = 0.511, p < 0.05) (Fig. S1a).

**Table 3:** Hierarchical analysis of AMOVA carried out using Cytb sequences and Rp S7 sequences to estimate levels of differentiation among groups of populations, among populations within groups and within populations.

Source of variation	Cytb			Rp S7		
	Variance components	Percentage of variation	Fixation index	Variance components	Percentage of variation	Fixation index
1. One gene pool						
Among populations	2.63669	73.95	F_ST_ = 0.739	0.75762	6.60	F_ST_ = 0.06604
Within populations	0.92867	26.05		10.71396	26.05	
2. Two gene pool						
Among populations	4.38825	82.48	F_SC_ = 0.00401	0.10350	0.90	F_SC_ = 0.06096
Among populations within groups	0.00374	0.07	F_ST_ = 0.82546	0.69552	6.04	F_ST_ = 0.06940
Within populations	0.92867	17.45	F_CT_ = 0.82476	10.71396	93.6	F_CT_ = 0.00899

**Figure 3:**
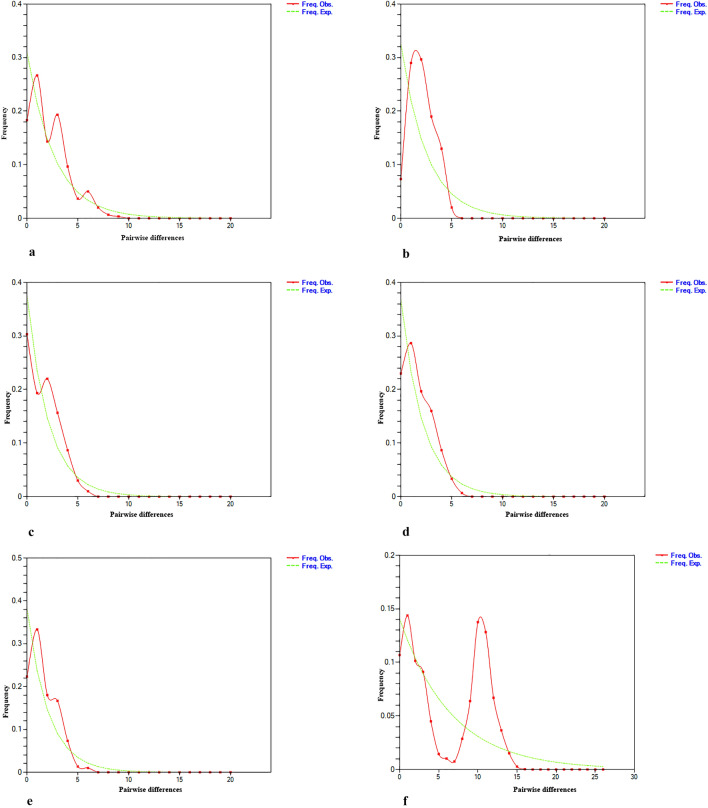
Mismatch distribution analysis using constant population size model to observe the population size changes in *N. randalli* populations based on Cytb gene sequences. The observed frequency represented by a red line. The expected frequency was depicted by a dotted green line.

The overall Tajima’s D (D =  − 1.1764, p > 0.05) and Fu’s Fs (Fs =  − 44.03, p < 0.05) values were negative, suggesting a deviation from neutrality. This negative value indicates an excess of rare haplotypes or rare mutations within populations. However, Tajima’s D value shows non-significant negative values. All five populations also had negative values for both Tajima’s D and Fu’s Fs and are statistically significant, suggesting population expansion (Table 4). The mismatch distribution graph for the entire population showed a bimodal and ragged pattern, indicating population equilibrium. However, individual populations showed both unimodal and bimodal patterns (Fig. 3). The mismatch distribution for east and west coasts showed unimodal and bimodal values respectively. The results of the mismatch distribution contradict the neutrality tests. Hence, to further test the validity of the neutrality test results, the raggedness index and SSD were calculated under the sudden demographic expansion model. The results showed that the data has a relatively good fit to a model of population expansion since both the Harpending Raggedness index and SSD values were non-significant for all populations, as shown in Table 4. In the Indian waters, the estimated tau (τ) values derived from the Cytb sequences were 1.57266 (Table 4). By applying the equation T = τ/2*u*, the resulting time for population expansion based on the Cytb sequences was determined to be 77,000 years. The estimated θ_1_ was higher than θ_0_, indicating population expansion from a small to a large size (Table 4). According to the Bayesian skyline plot (BSP), there was a demographic expansion approximately 77,000 years ago. Prior to this expansion, the flat skyline plot suggested that the population had remained stable with a constant size during that time period (Fig. S2).

**Table 4:** Demographic parameters of *N. randalli* based on Cytb sequences.

	Cochin	Chennai	Mangalore	Puri	Veraval	Mean
Neutrality tests						
Tajima’s D	− 2.095**	− 2.199**	− 1.779*	− 1.726*	− 2.079**	− 1.976
Fu’s Fs	− 5.706***	− 18.140***	− 7.662***	− 5.884***	− 9.654***	− 9.409
Mismatch distribution						
Demographic expansion						
τ	0.67188	2.14844	2.86719	1.31250	0.86328	1.57266
θ_0_	0.00000	0.00000	0.00000	0.75586	0.82969	0.31711
θ_1_	99,999.0	99,999.0	2.73618	6.38730	99,999.0	60,001.22
SSD	0.15468	0.00548	0.00373	0.00153	0.00486	0.03406
Hri	0.03888	0.07447	0.02543	0.02163	0.04821	0.04172
Spatial expansion						
τ	0.67320	2.14531	2.16729	0.90813	0.86574	1.35193
θ	1.76342	0.00258	0.00072	1.01028	0.82641	0.72068
SSD	0.00762	0.00548	0.00014	0.00166	0.00486	0.00396
Hri	0.03888	0.07447	0.02543	0.02163	0.04821	0.04172

*p < 0.05, **p < 0.01, ***p < 0.001.

### Nuclear DNA

An intron fragment 577 bp long was obtained from 125 individuals across five sampling locations, resulting in a total of 250 sequences. The average nucleotide composition was C: 20.93%, T: 30.90%, A: 22.19% and G: 25.99%. A total of 186 alleles were detected in all samples, with 293 segregating sites consisting of 17 singleton sites and 276 parsimony informative sites (Table S2). Of the 440 substitutions observed, 212 were transitions and 228 were transversions, and there were no shared alleles between populations. Overall allelic diversity was high (Ad = 0.997), but nucleotide diversity was low (π = 0.039). The number of alleles, allelic diversity, nucleotide diversity and the average number of nucleotide differences of individual populations are presented in Table 1. The median-joining haplotype network for Rp S7 gene intron 1 sequences revealed a complex pattern due to high levels of polymorphism, with a less obvious geographical structure compared to that detected for the cytb gene sequences (Fig. S3). The Bayesian tree topology did not reveal partition based on geographical region. No obvious structure was observed in the Rp S7 haplotypes from Indian coast (Fig. S4). Genetic distances within populations ranged from 2.5 to 4.9% and genetic distances between populations ranged from 2.9 to 5.2%. Since the nuDNA sequences show high polymorphism, it will reflect the overall genetic divergence. As a result, populations may exhibit higher K2P values (Table 2).

Pairwise F_ST_ for populations within the coasts showed non-significant structuring, with low F_ST_ and Φ_ST_ between all populations (Table 2). Hierarchical AMOVA based on two gene pool showed non-significant genetic differentiation (Φ_CT_ = 0.008, p = 0.195) (Table 3). The analysis conducted using SAMOVA indicated a non-significant result, suggesting that no genetic structure was detected in the population. The mantel test showed non-significant correlations between genetic and geographical distance where r = 0.447, p = 0.16 (Fig. S1b).

## Discussion

The present results provided insights into the patterns of genetic differentiation and demographic history of *N. randalli* in the Indian waters. Previous studies have investigated the population structure of *N. randalli* using the mt D-loop region^27^, the COI region^29^, and the otolith shape^28^. Srihari et al.^27^ revealed a lack of structuring among populations from Indian waters. These studies were conducted at a reduced geographical scale mainly along the west coast which may be the reason for the lack of significant genetic differentiation. In a separate study, Srihari et al.^28^ investigated the stock structure of *N. randalli* based on morphology and otolith shape from five major fishing regions along the Indian coast and observed different phenotypic stocks along the Indian coast. However, they concluded that the observed heterogeneity might be due to adaptation to the particular environmental conditions in this region.

The present work is characterized by high haplotype diversity and low nucleotide diversity for each population and across all populations as revealed by both mtDNA Cytb and nuDNA Rp S7 sequences suggesting a recent population expansion after a period of low effective size^30^. The presence of genetic differentiation among populations of *N. randalli* was confirmed through pair-wise F_ST_ and AMOVA analysis. The measurement of F_ST_ enables the assessment of genetic variation within and between populations, providing valuable insights into how evolutionary processes impact genetic composition^31^. Wright characterized F_ST_ values as follows: 0–0.05 indicates little differentiation, 0.05–0.15 indicates moderate differentiation, 0.15–0.25 indicates large differentiation, and values greater than 0.25 indicate very large differentiation^32^. When comparing the mitochondrial and nuclear markers to assess the genetic differentiation of *N. randalli*, the molecular analyzes performed led to contradictory results. In particular, the mitochondrial marker indicated high genetic differentiation, suggesting the presence of two distinct stocks, one on the east coast and one on the west coast. This was further supported by hierarchical AMOVA analysis based on Cytb DNA sequences, which showed significant F_ST_ and F_CT_ values, indicating significant genetic structure among *N. randalli* populations. In contrast, the analysis based on the intron 1 sequences of the Rp-S7 gene did not reveal any significant genetic differentiation in the hierarchical AMOVA. In this study, the F_ST_ values obtained for Cytb showed high genetic differentiation, suggesting the presence of two distinct populations, one on the east coast and one on the west coast. This structure was present in the Cytb results but not in the Rp S7 results. The median joining network for Rp-S7 gene intron 1 sequences represented an ambiguous haplotype network due to high polymorphism, indicating a less obvious geographical structure compared to the Cytb gene sequences.

The median joining network diagram based on Cytb gene sequences revealed the presence of two haplogroups in which individuals from the east coast were assigned to haplogroup I and from the west coast to haplogroup II. The high level of genetic differentiation among *N. randalli* populations in Indian waters, as observed in the Cytb DNA sequences, is corroborated by the corresponding phylogenetic tree. This tree exhibits results consistent with the hierarchical AMOVA, revealing two distinct clades with a high posterior probability value, indicating geographical differentiation among haplotypes. However, phylogenetic analyses of the nuclear Rp S7 gene intron 1 did not reveal significant genetic differentiation among alleles, suggesting a more uniform genetic composition for this marker. Furthermore, application of SAMOVA to the Cytb DNA sequences, unveiled a clear geographical separation among *N. randalli* populations along the Indian coast. In contrast, the SAMOVA results for the Rp S7 gene intron 1 sequences did not indicate significant genetic structure within the population, implying that this particular marker may not be as informative in discerning geographical differences among *N. randalli* populations. The lack of genetic differentiation as indicated by the Rp S7 gene intron 1 sequences is due to the high variability of intronic regions which masked the signals of genetic differentiation if any. Similar results of high intronic variability have been indicated in studies of species like the Amazonian cardinal tetra^33^.

Discrepancies are evident in the results obtained from mitochondrial DNA (mtDNA) and nuclear DNA (nuDNA) sequences. The mtDNA sequences reveal genetic differentiation between the east and west coast, characterized by the absence of haplotype sharing. In contrast, the nuDNA sequences show no genetic differentiation between the east and west coast populations. The efficiency of mitochondrial markers in detecting population differentiation is attributed to their lower effective population size^34^. Additionally, the faster genetic drift, coupled with a significantly higher mutation rate (about five to ten orders of magnitude) than that of nuclear DNA, enhances their discriminatory power^35^. The nuclear DNA, inherited from both parents, allows the examination of both maternal and paternal lineages, providing a comprehensive understanding. The biparental inheritance of nuDNA facilitates the interpretation of genetic lineages. The high polymorphism in nuclear markers is advantageous for revealing subtle population structures, aiding in identification of subpopulations. However, the extensive polymorphism can occasionally complicate the interpretation of population structure.

The genetic differentiation observed in *N. randalli* can be influenced by various life history traits exhibited by the species. Traits such as non-migratory behaviour, natal homing behaviour, parental care, characteristics of eggs, and limited larval dispersal have the potential to restrict gene flow between populations, leading to genetic differentiation. However, our understanding of these specific traits in *N. randalli* is currently limited. While it is known that *N. randalli* displays non-migratory behaviour and scatters its eggs on open substrates without guarding them^36^, information regarding the distribution and duration of the larval stage is lacking. The spawning season of this species is observed to be from June to October in Kerala^3^. This information is missing from other regions which constraints the interpretation of biological causes of genetic differentiation. The limited migratory capacity of *N. randalli* may contribute to the observed genetic differentiation between the east and west coasts.

The present study also indicated that *N. randalli* populations fits the isolation by distance model. The relationship between geographical and genetic distances showed significant scatter, indicating that there was considerable variation in the data. The Mantel test provides further evidence for the genetic structure observed in our study, suggesting that it aligns with the phenomenon commonly found in fish species with limited dispersal capabilities^37^. This also supports genetic differentiation between populations of *N. randalli.* In contrast, when examining Rp S7 gene intron 1 sequences, no significant correlation between genetic and geographic distances was observed. This lack of correlation suggests that there is no substantial evidence of genetic isolation by distance.

The genetic differentiation observed between *N. randalli* species on the west and east coasts of India could be due to a combination of factors including historical events, geographical barriers, and environmental differences. Geographical barriers such as ocean currents and physical features like mountain ranges or shallow water areas can also prevent gene flow between populations, leading to genetic differentiation. The Indian peninsula divides the Northern Indian Ocean into two marine regions, the Bay of Bengal on the east and Arabian Sea on the west which might restrict gene flow between these two coasts^38,39^. Another hard barrier which may restrict the gene flow between the west and east coasts of India is the Adam’s Bridge or Rama’s Bridge between Pamban Island, off the southeast coast of the southern Indian state of Tamil Nadu, and Mannar Island off the north west coast of Sri Lanka. This bridge formed a land barrier potentially preventing the spread of *N. randalli* contributing to the genetic differentiation^40^.

The Arabian Sea on the west and Bay of Bengal on the east coasts of India have different oceanographic conditions. The Arabian Sea exhibits higher productivity compared to the Bay of Bengal, with significant upwelling events during the summer monsoon^41^. The upwelling along the Arabian Sea is influenced by monsoonal winds and Ekman transport^42^. The prevailing monsoon winds blow parallel to the coastline, resulting in Ekman transport, that advects nutrient-rich water into the euphotic zone, promoting upwelling. This upwelling sustains cooler waters and supports abundant biological productivity, making the Arabian Sea one of the most productive regions in the Indian Ocean. In contrast, the upwelling in the Bay of Bengal is generally weaker and occurs primarily during the winter monsoon^43^. The south-westerly monsoon winds induce Ekman pumping and upwelling in the Bay of Bengal. The Bay of Bengal receives significant rainfall and freshwater from river runoff, leading to higher stratification and the formation of a thick barrier layer, inhibiting strong vertical mixing driven by summer monsoon winds (Ekman pumping)^44^. These contrasting oceanographic conditions serve as barriers to gene flow between fish populations on the west and east coasts of India.

Finally, environmental differences such as temperature, and salinity, can also influence genetic differentiation. The west and east coasts of India have different environmental conditions, with the west coast experiencing a more arid climate and higher salinity due to intrusion of high salinity waters from the Persian Gulf and the Red Sea. Additionally, it is the warmest region, with evaporation exceeding precipitation while the east coast has large amounts of precipitation influenced by monsoon rains and considerable influx of freshwater from major river systems leading to lower salinity^38,45,46^. The north Indian Ocean attains its highest global temperatures before the onset of the summer monsoon, specifically during the months of April and May^47^. During the south west monsoon, the Arabian Sea cools faster and sea surface temperature (SST) decreases than in the Bay of Bengal. While stratification associated with heavy precipitation and river run-off in the Bay of Bengal hampers the mixing of warm surface waters with cooler waters below, resulting in the Bay of Bengal retaining warmer SST than the Arabian Sea^48^. These environmental differences could lead to differences in the selection pressures acting on fish populations, leading to genetic differentiation. Overall, the genetic differentiation observed between some fish species on the west and east coasts of India is likely due to a combination of historical, geographical, and environmental factors. Other marine organisms have also been reported to exhibit a genetic structure that indicates genetic differentiation between the east and west coasts of India^49,50^.

Mismatch analyses revealed a bimodal distribution pattern, which is typically regarded as indicative of prolonged population stability^51^. Conversely, the values of Tajima’s D, Fu’s Fs, SSD, and Hri indicated that there was a demographic expansion. The multimodal mismatch distribution could also be attributed to population sub structuring^52,53^. Thus, we inferred that the bimodal pattern observed in the mismatch distributions described above is due to the existence of distinct haplogroups, rather than demographic stability. In contrast, each population exhibited a unimodal mismatch distribution pattern indicating that the population experience expansion. The mismatch distribution analysis conducted on the east coast population of *N. randalli* displayed a unimodal graph, indicating a signature of population expansion. In contrast, the mismatch distribution analysis performed on the west coast population revealed bimodal characteristics, suggesting that the population was in equilibrium, possibly due to a colonization event involving random haplotype lineages^54^.

The demographic expansion of *N. randalli* populations based on the Bayesian skyline plot and estimates of τ value from mismatch analysis indicated the time of expansion as 77,000 years ago during late Pleistocene, which was also supported by neutrality tests such as Tajima’s D and Fu’s Fs and mismatch distribution. During the late Pleistocene, which lasted from approximately 126,000 to 11,700 years ago, several climatic and environmental factors contributed to the expansion of marine populations. The late Pleistocene was marked by significant fluctuations in sea levels due to glacial-interglacial cycles^55^. During glacial periods, when large volumes of water were locked up in continental ice sheets, sea levels dropped, exposing vast areas of the continental shelves. As the Late Pleistocene progressed, the large continental ice sheets that had formed during the preceding glacial periods began to melt. The melting of ice sheets led to a significant increase in sea levels, flooding of coastal areas and expansion of marine habitats. This allowed marine populations to colonize newly available regions and contributed to their expansion. One factor that could contribute to distribution of *N. randalli* is historical events such as past changes in sea level or oceanographic conditions. The Last Glacial Maximum (LGM) occurred approximately 26,500 to 19,000 years ago, and had a significant impact on the North Indian Ocean. During this period, the Earth's climate was colder, and a large amount of water was trapped in ice sheets, resulting in lower sea levels worldwide^56^. Sea level decline leads to the formation of land bridges connecting Sri Lanka and India, affecting the dispersal of marine species and leading to the isolation of marine populations^40,57^. As a result, populations on the east and west coasts of India may have been separated for long periods of time. The Indian Ocean is influenced by the Indian monsoon system, characterized by seasonal winds and rainfall patterns^58,59^. During the Pleistocene, variations in monsoonal intensity occurred, affecting oceanic conditions. The strengthening of the Indian monsoon during the interglacial periods led to increased rainfall and freshwater runoff into the Indian Ocean. This freshwater influx, coupled with nutrient-rich sediment carried by rivers, enhanced productivity in coastal areas, supporting the growth and expansion of marine populations^60–62^. Oceanic currents and upwelling events influenced the distribution and abundance of marine populations in the Indian Ocean during the late Pleistocene. The Indian Ocean's circulation patterns, such as the Indian Ocean Dipole and the Somali Current, played a role in transporting nutrients and supporting the growth of marine life. Upwelling events, driven by wind patterns and oceanic circulation, brought nutrient-rich waters to the surface^63^, creating productive zones that fuelled the expansion of *N. randalli* populations.

## Conclusion

This study provides insights into the genetic structure and diversity of *N. randalli*. In our study, we showed evidence of significant population structuring in *N. randalli* populations using mitochondrial Cytb gene sequences. Mitochondrial DNA analysis of *N. randalli* showed genetic differentiation between the west and east coasts of India. The high level of polymorphism in the nuclear marker RpS7 masked the population structuring in *N. randalli*. Thus, concordant results were not obtained using mitochondrial and nuclear DNA sequences. In the present study, the nuclear marker showed a reduced efficiency to delineate populations with the given sample size. Hence, further studies should be carried out with advanced marker to delineate stock structure of *N. randalli*.

The high genetic structuring observed in *N. randalli* along the Indian waters indicates the necessity of devising distinct fishery management and conservation plans for the east and west coasts of India. Even though *N. randalli* is considered as a species of “least concern” by the IUCN, this does not mean that it is not endangered. By studying stock structure, such commercially important fish species can be managed in a way that maximizes economic benefits while minimizing negative impacts on the environment and the long-term health of the fish populations. This knowledge can contribute to a more comprehensive understanding of stock structure and inform conservation efforts more broadly.

## Materials and methods

### Molecular methods

#### Sampling and DNA extraction

A total of 125 samples of *N. randalli* were collected from five locations (Cochin (Coc), Chennai (Che), Mangalore (Man), Veraval (Ver) and Puri (Pur)) (Table S3) along Indian coast. Muscle tissues were collected from the caudal region (approximately 50 mg) and preserved in 95% ethanol and stored at room temperature. The fish samples used in this study were handled according to the guidelines for the care and use of fish in research by DeTolla et al.^64^. The protocols were approved by the Ethics Committee of ICAR—Central Marine Research Institute, Kochi. These methods are also reported in accordance with the ARRIVE guidelines (http://arriveguidelines.org). Total genomic DNA was extracted from tissues of *N. randalli* specimens using phenol–chloroform extraction method^65^ and stored at -20^0^C. The quantity and quality of DNA were measured by using Nanodrop (Thermo Fisher Scientific, USA) and integrity was determined using 0.8% agarose gel.

#### PCR amplification and sequencing

The partial mitochondrial Cytochrome b (Cytb, 507 bp) region and the single-copy ribosomal protein S7 gene intron 1 (Rp S7, 577 bp) were amplified using universal primers Cytb A-CCATGAGGACAAATATCATTYTG (Forward)^66^ and Cytb C-CTACTGGTTGTCCTCCGATTCAT (Reverse)^67^ and S7RPEX1F-TGGCCTCTTCCTTGGCCGTC and S7RPEX2R-AACTCGTCTGGCTTTTCGCC^68^ respectively.

For both the markers, PCR amplification was performed in a total volume of 20 µl reaction mixture containing 1 µl template DNA (50 ng), 0.4 µl of each forward and reverse primer, 8.2 µl PCR water and 10 µl master mix (TaKaRa) using a Biorad T100 thermocycler (Biorad, USA). The PCR program for the Cytb region involved an initial denaturation at 94 °C for 4 min, followed by 32 cycles of 94 °C denaturation for 30 s, 55 °C annealing for 30 s and 72 °C extension for 1 min, followed by a final extension at 72 °C for 10 min and a final hold at 4 °C. The PCR program for Rp S7 gene intron 1 involved an initial denaturation at 94 °C for 4 min, followed by 32 cycles of 94 °C denaturation for 30 s, 58 °C annealing for 30 s and 72 °C extension for 1 min, followed by a final extension at 72 °C for 10 min and a final hold at 4 °C. PCR products obtained were subjected to electrophoresis on 1.2% agarose gel stained with ethidium bromide and visualized using gel documentation system (Vilber Lourmat, France). The PCR products were then sequenced using Sanger sequencing.

### Sequence analysis

All sequences were checked manually against their chromatogram using the program Seqman from the software DNAStar Lasergene 6.0 (DNASTAR Inc., Madison, WI, USA). Sequences were manually checked, assembled, aligned and trimmed using Clustal W implemented in MEGA 7.0 software^69^. All the mitochondrial DNA sequences (Cytb) were translated into aminoacids using MEGA 7.0 software to ensure that stop codons were not present^69^. Heterozygous positions in individual sequences of nuclear genes were initially checked for double peaks in DNAStar Lasergene 6.0 software and alleles of the heterozygous individuals were phased using Bayesian program PHASE implemented in DnaSP 6.0^70^. The runs comprised 1000 number of iterations and 1000 burn-in iterations. The phased data were then used for the diversity analysis and network construction. The sequences were verified as mtDNA Cyt b region and ribosomal protein S7 region through BLAST in the NCBI. All unique haplotypes and alleles were deposited in the GenBank database under accession no OR178681–OR178738 (Cyt b) and OR180116–OR180301 (Rp S7).

### Statistical analysis

#### Diversity analysis

Genetic diversity indices such as number of haplotypes (h) or alleles (for nuclear), haplotype diversity (H_d_) or Allelic diversity (A_d_) (for nuclear), nucleotide diversity (π), number of segregating sites (S), average number of nucleotide differences (k), singleton and parsimony informative sites, were estimated for each and entire population using DnaSP 6.0^70^ and Arlequin v3.5^71^ for both data sets. The complete aligned data set was also assessed for nucleotide composition, transition and transversion using Arlequin v3.5^71^.

#### Historical demography

Population demographic history (Cytb) was assessed using neutrality tests and mismatch distribution analysis (MDA) implemented in DnaSP 6.0^70^ and Arlequin v3.5^71^. Neutrality tests such as Tajima’s D^72^ and Fu’s Fs^73^ were conducted to assess whether the DNA sequences experienced any deviation from neutral mutation hypothesis. Mismatch distribution was done to test whether a population experienced any expansion event. The calculated expected values for a model with a constant population size were plotted against the observed values. Populations that have undergone expansion are expected to have unimodal distribution, whereas a population at equilibrium gives multimodal distribution^51^. In addition, goodness of fit was assessed by the sum of squared deviation (SSD)^51^ and Harpending Raggedness index (Hri)^74^ between the observed and expected distribution under models of demographic and spatial expansion and its significance was determined by a parametric bootstrap with 10,000 replicates. The expansion signal for a population was indicated by smooth distribution with non-significant p values. The distribution of pairwise differences between sequences (mismatch distribution) were also estimated based on the demographic parameters θ_0_ (population size before expansion), θ_1_ (population size after expansion) and τ (time since expansion expressed in units of mutational time) using a generalized least-square approach implemented in Arlequin v3.5^71^. The time since last population expansion (T) was calculated using equation T = τ/2*u*^51^. The parameter *u* can be measured as *u* = µk, where µ is the mutation rate per nucleotide which is 2% per million years (Myr) for mitochondrial Cytb region of fishes^75^ with an estimated generation time of 1 year and k is the number of nucleotides covered in the data (507 bp). Coalescent-based Bayesian skyline plot (BSP) approach^76^ was used to estimate changes in effective population size (Ne) through time using BEAST software v1.8^77^. The analysis was run for 10^8^ iterations with burn-in of 10^7^ under HKY model for cytb gene sequences. A strict molecular clock and stepwise skyline model were applied. The model parameters and genealogies were sampled every 1000 iterations, and all operations were automatically optimized. Finally, the resulting skyline plot was visualized using Tracer v1.5^78^.

#### Population genetic structure

The pairwise genetic divergence within and among populations were calculated by MEGA 7^69^ using Kimura 2-parameter model^79^. Pairwise genetic divergence between population were computed using fixation index (F_ST_) and their significance was assessed with 10,000 permutations using Arlequin v3.5^71^. The significance of pairwise comparisons was adjusted by sequential Bonferroni corrections^80^. After correcting with Bonferroni corrections, significant probability values were reported for pairwise comparisons of F_ST_ values between all populations as well as among subpopulations. Analysis of molecular variance (AMOVA)^81^ was performed for both data sets to detect genetic differentiation among and between populations with 10,000 permutations to examine the statistical significance of fixation indices using Arlequin v 3.5^71^. In addition to the global AMOVA (considering all population-single gene pool) (global Φ_ST_), a hierarchical AMOVA was carried out to estimate relative contribution of molecular variance among groups (Φ_CT_), among populations within group (Φ_SC_) and among populations relative to total samples (Φ_ST_) with two gene pool (west: Cochin, Mangalore, Veraval; east: Chennai, Puri). Spatial Analysis of Molecular Variance (SAMOVA) v.2.0^82^ was utilized to further investigate the spatial structure, aiming to identify groups of populations that exhibited geographical homogeneity while displaying the highest level of differentiation from one another. The value of K (no. of groups) was set between 2 and 5, with 10,000 simulated annealing steps. The optimal number of population groups was determined based on the highest variance among groups (F_CT_). Isolation by Distance (IBD) was tested to understand whether there is a correlation between genetic distance (F_ST_ from Arlequin) and geographical distance (km) using the Mantel test^83^ with 10,000 permutations implemented in Arlequin v3.5^71^. Geographic distances between sampling sites were obtained using Google Earth v.9 based on longitude and latitude. To interpret and visualize phylogeographic relationship among haplotypes or alleles (for nuclear), median-joining haplotype network was constructed using PopART^84^.

#### Phylogenetic analysis

Phylogenetic analyses were carried out on the Cytb and S7 gene sequences separately to investigate the relationships among haplotypes/alleles from five geographical locations. The Bayesian inference method was used for both mitochondrial DNA (mtDNA) and nuclear DNA (nuDNA) to construct the phylogenetic tree. MrBayes v 3.2.4 was used to perform these analyses^85^. The best nucleotide substitution model for constructing the phylogenetic tree for each marker was inferred using MEGA 7 based on the AIC (Akaike Information Criterion)^69^. *Spondyliosoma cantharus* was chosen as the outgroup (GenBank accession no: MH545766 (Cytb) and MH545829 (Rp S7)). GTR + G + I was selected as the best model for constructing the phylogenetic tree for mtDNA and nuDNA. The Bayesian analysis was run for 1,000,000 generations using the Marko chain Monte Carlo (MCMC) method, with sampling done every 1000 generations. The analysis was stopped when the average standard deviation of split frequency was less than 0.01. The first 25% of the results were discarded as burn-in. The resulting phylogenetic tree was viewed using FigTree v1.4.3 (http://tree.bio.ed.ac.uk/software/figtree/).

### Supplementary Information

Supplementary Information.

## Data Availability

All data generated or analyzed during this study is included in this published article (and its supplementary files). In addition, the sequence data generated in this study is available in the NCBI-GenBank under the accession numbers: OR178681–OR178738 and OR180116–OR180301 (https://www.ncbi.nlm.nih.gov/genbank/).
