# Supplementary material for: Population genetic structure of Randall’s threadfin bream Nemipterus randalli in Indian waters based on mitochondrial and nuclear gene sequences
Source: Sci Rep. 2024 Mar 30;14:7556. doi: 10.1038/s41598-024-58047-1 (PMC10981751; doi:10.1038/s41598-024-58047-1)
Supplement: Supplementary file 1 — Supplementary Information. [file 41598_2024_58047_MOESM1_ESM.docx]

**POPULATION GENETIC STRUCTURE OF RANDALL’S THREADFIN BREAM *NEMIPTERUS RANDALLI* IN INDIAN WATERS BASED ON MITOCHONDRIAL AND NUCLEAR GENE SEQUENCES**

**Author affiliations**

**Neenu Raj^a, b, *^, Sandhya Sukumaran^a^, Anjaly Jose^a^, Nisha K^a^, Subal Kumar Roul^a^, Shikha Rahangdale^a^, Shoba Joe Kizhakudan^a^ & A. Gopalakrishnan^a^**

**^a^Marine Biotechnology Fish Nutrition and Health Division, ICAR-Central Marine Fisheries Research Institute, Ernakulam North P.O., Kochi, Kerala, India – 682018**

**^b^Mangalore University, Mangalagangotri, Mangalore, Karnataka, India - 574 199**

***Corresponding author**

Correspondence to Neenu Raj

E-mail address: neenuraj993@gmail.com; Ph. No: +91-9496501388

**Supplementary files**

**Table S1. Distribution of Cytb haplotypes in *N. randalli* among the five locations**

| Location | Haplotype | | | | | | | | | | | | | | | | | | | |
| --- | --- | --- | --- | --- | --- | --- | --- | --- | --- | --- | --- | --- | --- | --- | --- | --- | --- | --- | --- | --- |
|  | 1 | 2 | 3 | 4 | 5 | 6 | 7 | 8 | 9 | 10 | 11 | 12 | 13 | 14 | 15 | 16 | 17 | 18 | 19 | 20 |
| Chennai |  |  |  |  |  |  |  |  |  |  |  |  | 7 | 1 | 1 | 1 | 1 | 1 | 1 | 1 |
| Cochin | 10 | 1 | 1 | 5 | 1 | 1 | 1 | 1 | 1 | 1 | 1 | 1 |  |  |  |  |  |  |  |  |
| Mangalore | 14 |  |  | 1 |  |  |  |  |  |  |  |  |  |  |  |  |  |  |  |  |
| Puri |  |  |  |  |  |  |  |  |  |  |  |  | 12 |  | 2 |  |  |  |  | 2 |
| Veraval | 12 |  |  |  |  |  |  |  |  |  |  |  |  |  |  |  |  |  |  |  |
|  | 21 | 22 | 23 | 24 | 25 | 26 | 27 | 28 | 29 | 30 | 31 | 32 | 33 | 34 | 35 | 36 | 37 | 38 | 39 | 40 |
| Chennai | 1 | 1 | 1 | 1 | 1 | 2 | 1 | 1 | 1 | 1 |  |  |  |  |  |  |  |  |  |  |
| Cochin |  |  |  |  |  |  |  |  |  |  |  |  |  |  |  |  |  |  |  |  |
| Mangalore |  |  |  |  |  |  |  |  |  |  | 1 | 1 | 1 | 1 | 1 | 1 | 1 | 1 | 1 | 1 |
| Puri |  |  |  |  |  |  |  |  |  |  |  |  |  |  |  |  |  |  |  |  |
| Veraval |  |  |  |  |  |  |  |  |  |  |  |  |  |  |  | 2 | 1 |  |  |  |
|  | 41 | 42 | 43 | 44 | 45 | 46 | 47 | 48 | 49 | 50 | 51 | 52 | 53 | 54 | 55 | 56 | 57 | 58 |  |  |
| Chennai |  |  |  |  |  |  |  |  |  |  |  |  |  |  |  |  |  |  |  |  |
| Cochin |  |  |  |  |  |  |  |  |  |  |  |  |  |  |  |  |  |  |  |  |
| Mangalore |  |  |  |  |  |  |  |  |  |  |  |  |  |  |  |  |  |  |  |  |
| Puri | 1 | 1 | 1 | 1 | 2 | 1 | 1 | 1 |  |  |  |  |  |  |  |  |  |  |  |  |
| Veraval |  |  |  |  |  |  |  |  | 1 | 1 | 1 | 1 | 1 | 1 | 1 | 1 | 1 | 1 |  |  |

**Table S2. Distribution of S7 alleles in *N. randalli* among the five locations**

| Location | Alleles | | | | | | | | | | | | | | | | | | | |
| --- | --- | --- | --- | --- | --- | --- | --- | --- | --- | --- | --- | --- | --- | --- | --- | --- | --- | --- | --- | --- |
|  | 1 | 2 | 3 | 4 | 5 | 6 | 7 | 8 | 9 | 10 | 11 | 12 | 13 | 14 | 15 | 16 | 17 | 18 | 19 | 20 |
| Chennai |  |  |  |  |  |  |  |  |  |  |  |  |  |  |  |  |  |  |  |  |
| Cochin | 2 | 1 | 1 | 1 | 1 | 1 | 1 | 2 | 2 | 1 | 1 | 2 | 1 | 1 | 1 | 1 | 1 | 1 | 1 | 1 |
| Mangalore |  |  |  |  |  |  |  |  |  |  |  |  |  |  |  |  |  |  |  |  |
| Puri |  |  |  |  |  |  |  |  |  |  |  |  |  |  |  |  |  |  |  |  |
| Veraval |  |  |  |  |  |  |  |  |  |  |  |  |  |  |  |  |  |  |  |  |
|  | 21 | 22 | 23 | 24 | 25 | 26 | 27 | 28 | 29 | 30 | 31 | 32 | 33 | 34 | 35 | 36 | 37 | 38 | 39 | 40 |
| Chennai |  |  |  |  |  |  |  |  |  |  |  |  |  |  |  |  |  |  |  |  |
| Cochin | 1 | 1 | 2 | 2 | 1 | 1 | 1 | 1 | 1 | 1 | 1 | 1 | 1 | 1 | 2 | 1 | 1 | 1 | 1 | 2 |
| Mangalore |  |  |  |  |  |  |  |  |  |  |  |  |  |  |  |  |  |  |  |  |
| Puri |  |  |  |  |  |  |  |  |  |  |  |  |  |  |  |  |  |  |  |  |
| Veraval |  |  |  |  |  |  |  |  |  |  |  |  |  |  |  |  |  |  |  |  |
|  | 41 | 42 | 43 | 44 | 45 | 46 | 47 | 48 | 49 | 50 | 51 | 52 | 53 | 54 | 55 | 56 | 57 | 58 | 59 | 60 |
| Chennai |  | 2 | 2 | 2 | 2 | 1 | 1 | 2 | 2 | 1 | 1 | 2 | 1 | 1 | 2 | 1 | 1 | 2 | 1 | 1 |
| Cochin | 2 |  |  |  |  |  |  |  |  |  |  |  |  |  |  |  |  |  |  |  |
| Mangalore |  |  |  |  |  |  |  |  |  |  |  |  |  |  |  |  |  |  |  |  |
| Puri |  |  |  |  |  |  |  |  |  |  |  |  |  |  |  |  |  |  |  |  |
| Veraval |  |  |  |  |  |  |  |  |  |  |  |  |  |  |  |  |  |  |  |  |
|  | 61 | 62 | 63 | 64 | 65 | 66 | 67 | 68 | 69 | 70 | 71 | 72 | 73 | 74 | 75 | 76 | 77 | 78 | 79 | 80 |
| Chennai | 2 | 1 | 1 | 2 | 1 | 1 | 2 | 1 | 1 | 2 | 1 | 1 | 1 | 1 | 1 | 1 | 1 | 1 |  |  |
| Cochin |  |  |  |  |  |  |  |  |  |  |  |  |  |  |  |  |  |  |  |  |
| Mangalore |  |  |  |  |  |  |  |  |  |  |  |  |  |  |  |  |  |  | 1 | 1 |
| Puri |  |  |  |  |  |  |  |  |  |  |  |  |  |  |  |  |  |  |  |  |
| Veraval |  |  |  |  |  |  |  |  |  |  |  |  |  |  |  |  |  |  |  |  |
|  | 81 | 82 | 83 | 84 | 85 | 86 | 87 | 88 | 89 | 90 | 91 | 92 | 93 | 94 | 95 | 96 | 97 | 98 | 99 | 100 |
| Chennai |  |  |  |  |  |  |  |  |  |  |  |  |  |  |  |  |  |  |  |  |
| Cochin |  |  |  |  |  |  |  |  |  |  |  |  |  |  |  |  |  |  |  |  |
| Mangalore | 2 | 1 | 1 | 2 | 1 | 1 | 2 | 2 | 2 | 2 | 1 | 1 | 2 | 1 | 1 | 1 | 1 | 1 | 1 | 2 |
| Puri |  |  |  |  |  |  |  |  |  |  |  |  |  |  |  |  |  |  |  |  |
| Veraval |  |  |  |  |  |  |  |  |  |  |  |  |  |  |  |  |  |  |  |  |
|  |  |  |  |  |  |  |  |  |  |  |  |  |  |  |  |  |  |  |  |  |
| Location | Alleles | | | | | | | | | | | | | | | | | | | |
|  | 101 | 102 | 103 | 104 | 105 | 106 | 107 | 108 | 109 | 110 | 111 | 112 | 113 | 114 | 115 | 116 | 117 | 118 | 119 | 120 |
| Chennai |  |  |  |  |  |  |  |  |  |  |  |  |  |  |  |  |  |  |  |  |
| Cochin |  |  |  |  |  |  |  |  |  |  |  |  |  |  |  |  |  |  |  |  |
| Mangalore | 2 | 2 | 2 | 1 | 1 | 1 | 1 | 2 | 1 | 1 | 2 | 2 | 2 |  |  |  |  |  |  |  |
| Puri |  |  |  |  |  |  |  |  |  |  |  |  |  | 2 | 1 | 1 | 1 | 1 | 2 | 2 |
| Veraval |  |  |  |  |  |  |  |  |  |  |  |  |  |  |  |  |  |  |  |  |
|  | 121 | 122 | 123 | 124 | 125 | 126 | 127 | 128 | 129 | 130 | 131 | 132 | 133 | 134 | 135 | 136 | 137 | 138 | 139 | 140 |
| Chennai |  |  |  |  |  |  |  |  |  |  |  |  |  |  |  |  |  |  |  |  |
| Cochin |  |  |  |  |  |  |  |  |  |  |  |  |  |  |  |  |  |  |  |  |
| Mangalore |  |  |  |  |  |  |  |  |  |  |  |  |  |  |  |  |  |  |  |  |
| Puri | 2 | 2 | 2 | 2 | 1 | 1 | 1 | 1 | 1 | 1 | 2 | 1 | 1 | 1 | 1 | 2 | 1 | 1 | 2 | 2 |
| Veraval |  |  |  |  |  |  |  |  |  |  |  |  |  |  |  |  |  |  |  |  |
|  | 141 | 142 | 143 | 144 | 145 | 146 | 147 | 148 | 149 | 150 | 151 | 152 | 153 | 154 | 155 | 156 | 157 | 158 | 159 | 160 |
| Chennai |  |  |  |  |  |  |  |  |  |  |  |  |  |  |  |  |  |  |  |  |
| Cochin |  |  |  |  |  |  |  |  |  |  |  |  |  |  |  |  |  |  |  |  |
| Mangalore |  |  |  |  |  |  |  |  |  |  |  |  |  |  |  |  |  |  |  |  |
| Puri | 2 | 2 | 2 | 2 | 2 | 1 | 1 |  |  |  |  |  |  |  |  |  |  |  |  |  |
| Veraval |  |  |  |  |  |  |  | 1 | 1 | 1 | 1 | 1 | 1 | 2 | 2 | 1 | 1 | 2 | 2 | 1 |
|  | 161 | 162 | 163 | 164 | 165 | 166 | 167 | 168 | 169 | 170 | 171 | 172 | 173 | 174 | 175 | 176 | 177 | 178 | 179 | 180 |
| Chennai |  |  |  |  |  |  |  |  |  |  |  |  |  |  |  |  |  |  |  |  |
| Cochin |  |  |  |  |  |  |  |  |  |  |  |  |  |  |  |  |  |  |  |  |
| Mangalore |  |  |  |  |  |  |  |  |  |  |  |  |  |  |  |  |  |  |  |  |
| Puri |  |  |  |  |  |  |  |  |  |  |  |  |  |  |  |  |  |  |  |  |
| Veraval | 1 | 1 | 1 | 1 | 1 | 2 | 1 | 1 | 1 | 1 | 1 | 1 | 2 | 1 | 1 | 2 | 1 | 1 | 1 | 1 |
|  | 181 | 182 | 183 | 184 | 185 | 186 |  |  |  |  |  |  |  |  |  |  |  |  |  |  |
| Chennai |  |  |  |  |  |  |  |  |  |  |  |  |  |  |  |  |  |  |  |  |
| Cochin |  |  |  |  |  |  |  |  |  |  |  |  |  |  |  |  |  |  |  |  |
| Mangalore |  |  |  |  |  |  |  |  |  |  |  |  |  |  |  |  |  |  |  |  |
| Puri |  |  |  |  |  |  |  |  |  |  |  |  |  |  |  |  |  |  |  |  |
| Veraval | 2 | 2 | 2 | 2 | 1 | 1 |  |  |  |  |  |  |  |  |  |  |  |  |  |  |
|  |  |  |  |  |  |  |  |  |  |  |  |  |  |  |  |  |  |  |  |  |

**Table S3. Sampling location and sample size of *N. randalli***

| **Region** | **Population** | **Latitude** | **Longitude** | **No: of samples** |
| --- | --- | --- | --- | --- |
| West coast | Cochin | 9^0^58’00.0’’N | 76^0^14’00.0’’E | 25 |
|  | Mangalore | 12^0^52’00.0’’N | 74^0^53’00.0’’E | 25 |
|  | Veraval | 20^0^54’00.0’’N | 70^0^22’00.0’’E | 25 |
| East coast | Chennai | 80^0^16’00.0’’N | 80^0^16’42.5’’E | 25 |
|  | Puri | 85^0^51’00.0’’N | 85^0^51’00.0’’E | 25 |

**a)**

**b)**

**Fig. S1 Isolation–by–distance in *N. randalli* populations. Genetic distances (F_ST_/1- F_ST_) based on a) Cytb and b) Rp S7 sequences data were plotted against geographical distances between five locations**


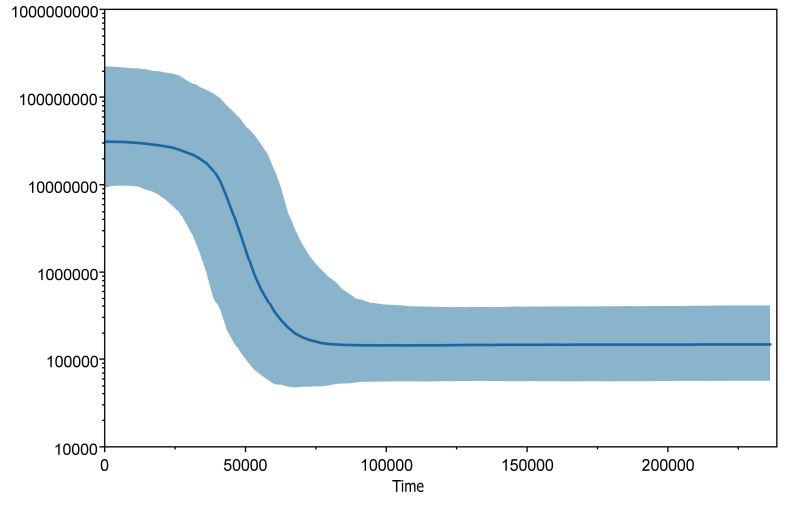


**Fig. S2 Bayesian skyline plot based on Cytb sequences of *N. randalli*. The X-axis indicates time in thousands of years before present and Y-axis represents effective population size. The thick line represents the mean estimate of effective population size. The two blue lines are the upper and lower bounds of the 95% HPD interval**

**
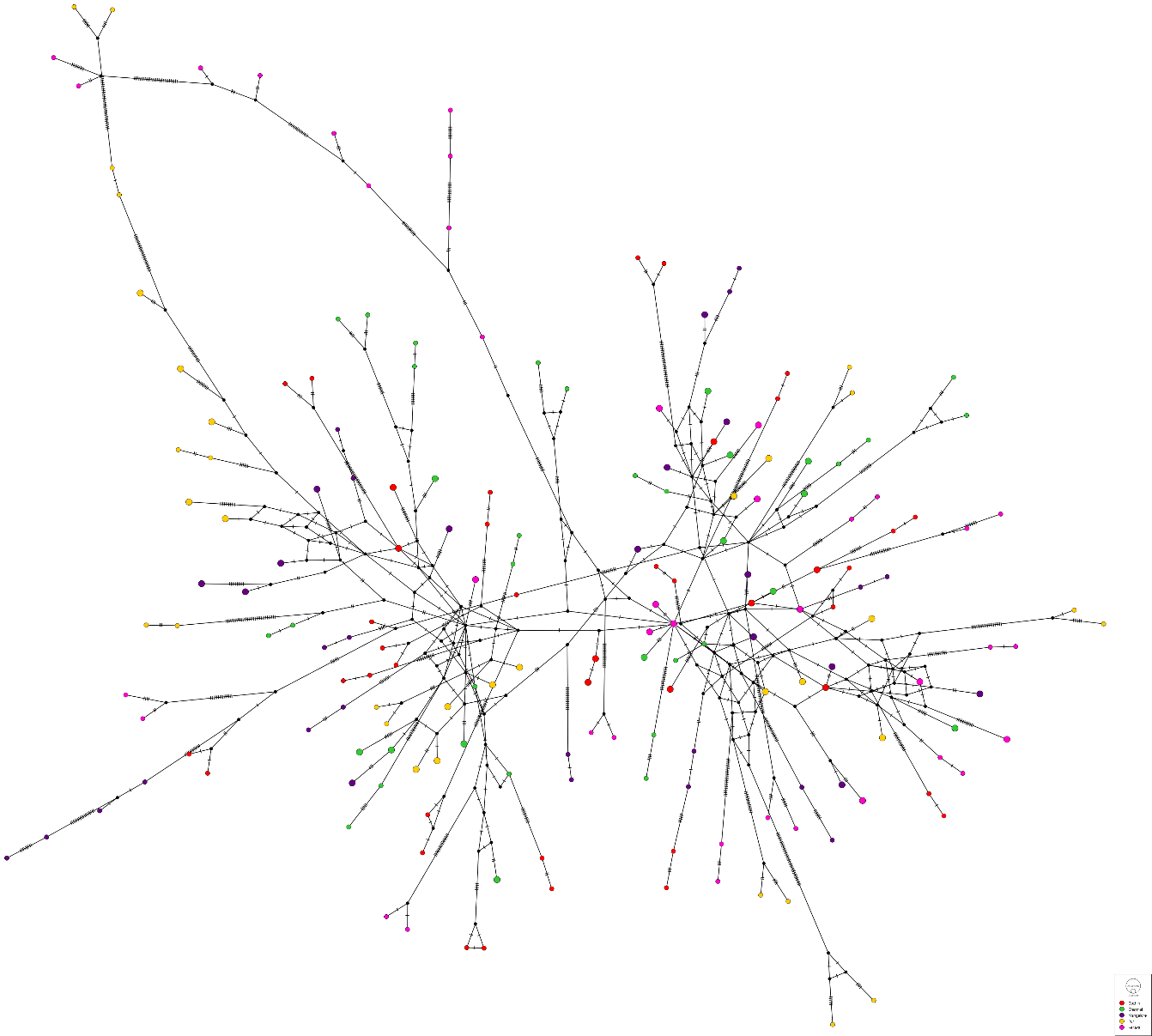
Fig. S3 Median joining network for *N. randalli* based on Rp S7 sequences. Alleles are represented in circles and colours indicate geographical locations. Vertical lines indicate mutational steps. Black dots indicate lost or not sampled alleles**


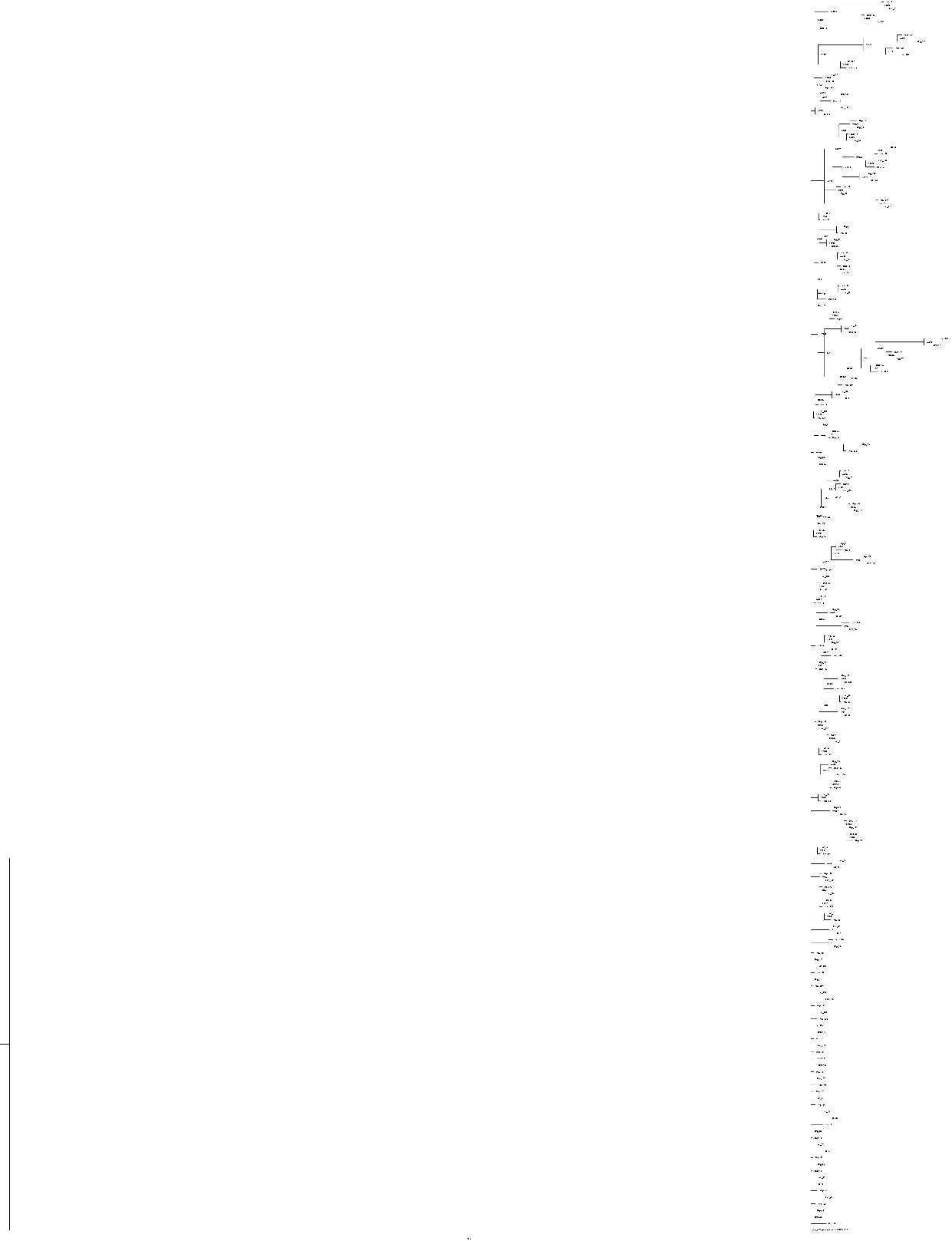


**Fig. S4 Bayesian tree based on Rp S7 sequences of *N. randalli.* Posterior probability values are shown at the nodes.**
